# Histopathology of COVID-19 pneumonia in two non-oncological, non-hospitalised cases as a reliable diagnostic benchmark

**DOI:** 10.1186/s13000-020-00990-4

**Published:** 2020-06-09

**Authors:** Roberto Scendoni, Francesca Marchesani, Nunzia Cannovo, Piergiorgio Fedeli, Mariano Cingolani

**Affiliations:** 1 Institute of Legal Medicine, Department of Law, University of Macerata, Piaggia dell’Università, 2, 62100 Macerata, Italy; 2 Pulmonary Unit ASUR Marche AV3, Via Santa Lucia, 62100 Macerata, Italy; 3 Institute of Legal Medicine – School of Law, University of Camerino, Via Andrea D’Accorso n. 16, 62032 Camerino, MC Italy

**Keywords:** COVID-19 pneumonia, Histopathology, Diffuse alveolar damage, Oncological patient, Lung cancer

## Abstract

In lung cancer patients infected with COVID-19, pathological features are not easy to distinguish. This report presents detailed histopathological findings in two non-neoplastic subjects whose out-of-hospital deaths were caused by COVID-19 infection. These ‘pure’ cases differ in the time of presentation of symptoms, the phase of lung anatomopathological patterns (acute lung injury versus diffuse alveolar damage) and the mechanism of death. The results provide a valid diagnostic benchmark for evaluating the evolution of COVID-19 pneumonia.

## Introduction

Although several months have passed since the epidemic spread of the virus, current information on the pathological characteristics of deaths caused by COVID-19 is often variable [1], and in neoplastic patients, especially those with lung cancer, these characteristics can overlap or be difficult to attribute correctly.

Authors [2] have recently considered lung disease in hospitalised patients with lung cancer who have also been infected with COVID-19. However, what is the borderline between cancer-related lung disease (whether the subject is undergoing therapy or not) and lung disease linked to COVID-19? It has been documented that oncological patients who develop an acute respiratory distress syndrome (ARDS) have a significantly higher risk of death compared with those who develop ARDS without cancer, and this increased risk appears to be correlated with the increased severity of illness at presentation [3].

In addition, can the presence of other infections besides COVID-19 be entirely ruled out? Studies have shown that many infectious agents are responsible for approximately 90% of all primary and secondary causes of ARDS in the cancer setting [4]. Furthermore, ARDS is a major cause of postoperative respiratory failure, with mortality rates reaching 40% in the general population. Mortality rates in the cancer population vary widely, depending on the type of cancer, and thoracic surgeries carry the highest operational risk of lung damage [5]. Plus, the case fatality rate is significantly higher for those with an underlying concomitant disease, such as cardiovascular disease, diabetes, or chronic respiratory disease. Lung cancer represents a specific scenario of cumulative risk factors for COVID-19 complications [6], although the clinical course for these patients is still not well defined.

In order to contribute to the body of knowledge in this area, we have presented the detailed pathological findings in two cases of non-neoplastic subjects whose out-of-hospital deaths were caused by COVID-19 infection. They differed in the time of presentation of symptoms and consequently manifested different anatomopathological patterns, the first defining an early phase of the infection and the second showing a later stage. It is hoped that these findings will contribute to a better description of the pulmonary pathology of Sars-Cov-2 and also improve treatment choices for all COVID-19 cases including cancer patients, in line with proposals by other authors.

## Cases and methods

Anamnestic data and the circumstances of the deaths of the subjects, who both died at home, were collected. Before autopsy an oropharyngeal swab for SARS-CoV-2 viral RNA was performed, which tested positive in both cases.

*Case* 1: a 44-year-old man affected by polymyositis without pharmacological treatment, who for 3 days presented fever, marked asthenia and dyspnoea, followed by progressive cardiorespiratory insufficiency until exitus.

*Case* 2: a 62-year-old woman suffering from hypertension (not undergoing pharmacological treatment), autoimmune hypothyroidism and type 2 diabetes treated with oral hypoglycaemics, deceased after 8–9 days of high fever and dyspnea.

### Methods for histopathology

Tissue samples were fixed in 4% formalin, processed, embedded in paraffin and then cut with a microtome to obtain two sets of thin sections of 8 μm. The sections obtained were first stretched in distilled water at ambient temperature to eliminate any folds, then in water at 40–45 °C and finally affixed to the glass slide. One set of sections was then dried in an oven at 37–40 °C and hematoxylin-eosin (HE) stained. The sections obtained were analyzed by light microscopy; magnified images (× 200 and × 400) were acquired of the HE stains with a high-resolution colour microscope camera.

*Method for detection of SARS-CoV-2 viral RNA.*

A naso-oropharyngeal swab kit for COVID-19 sample collection and transport was used. Using a Real-time PCR thermal cycler, SARS-CoV-2 viral RNA was tested for and detected in both cases.

## Results

### Case 1

#### Autopsy

Macroscopic findings included the presence of extremely warm organs and dense blood. The heart examination revealed thrombosis of the anterior descending coronary artery.

When the thoracic cavity was opened, the lungs failed to collapse and there was a lack of visible exudates in airways. Both lungs had a thicker consistency (case 2 more than case 1), were compact, congested and edematous (case 1 more than case 2).

#### Microscopic findings

**Fig. 1:**
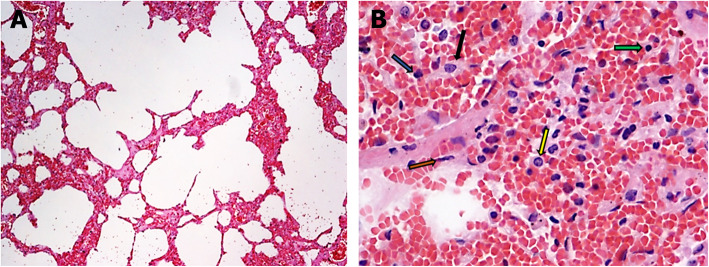
Histological changes in case 1. **a** Exudative phase of acute interstitial pneumonia (hematoxylin and eosin stain, × 100). **b** Detail of an alveolar septum with edema, hemorrhage and inflammatory cell infiltrations (green arrow: lymphocyte; yellow arrow: macrophage; brown arrow: fibroblast; blue arrow: polymorphonuclear leukocyte; black arrow: type II hyperplastic pneumocyte) (hematoxylin and eosin stain, × 400)

Thickening of the alveolar septa because of edema, hemorrhage and inflammatory cell infiltrations in the interalveolar septa (Fig. 1a). Mild presence of type II hyperplastic pneumocytes and fibroblasts in the alveolar septa (Fig. 1)b.

### Case 2

#### Autopsy

As in case 1, the organs were extremely warm and the blood was particularly dense. Macroscopic examination revealed intracardiac thrombosis and bilateral massive thromboembolism. Both lungs were markedly edematous and compact.

#### Microscopic findings

**Fig. 2:**
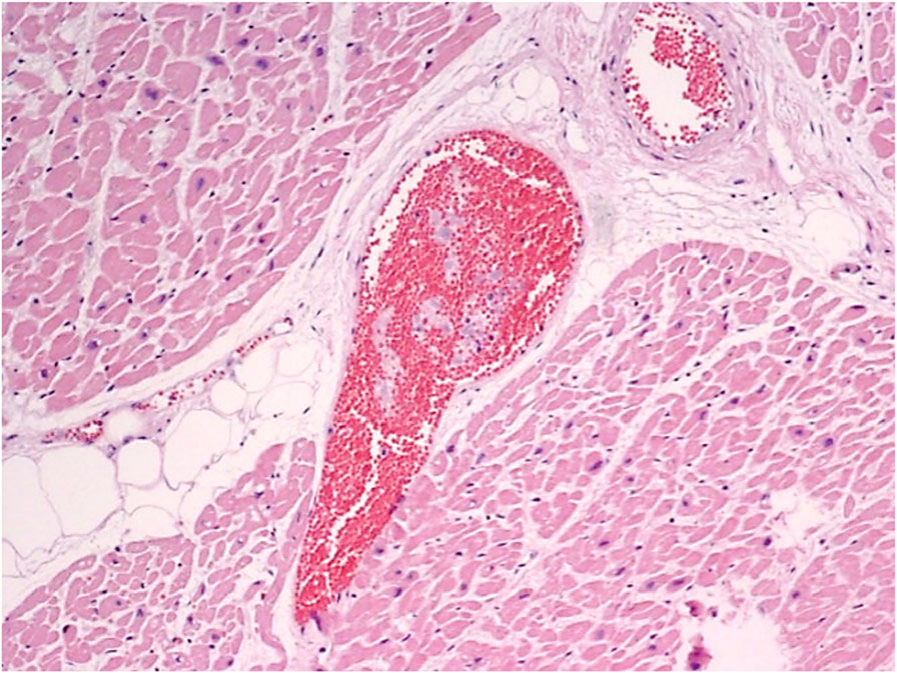
Myocardial vascular microthrombosis (hematoxylin and eosin stain, × 200)

**Fig. 3:**
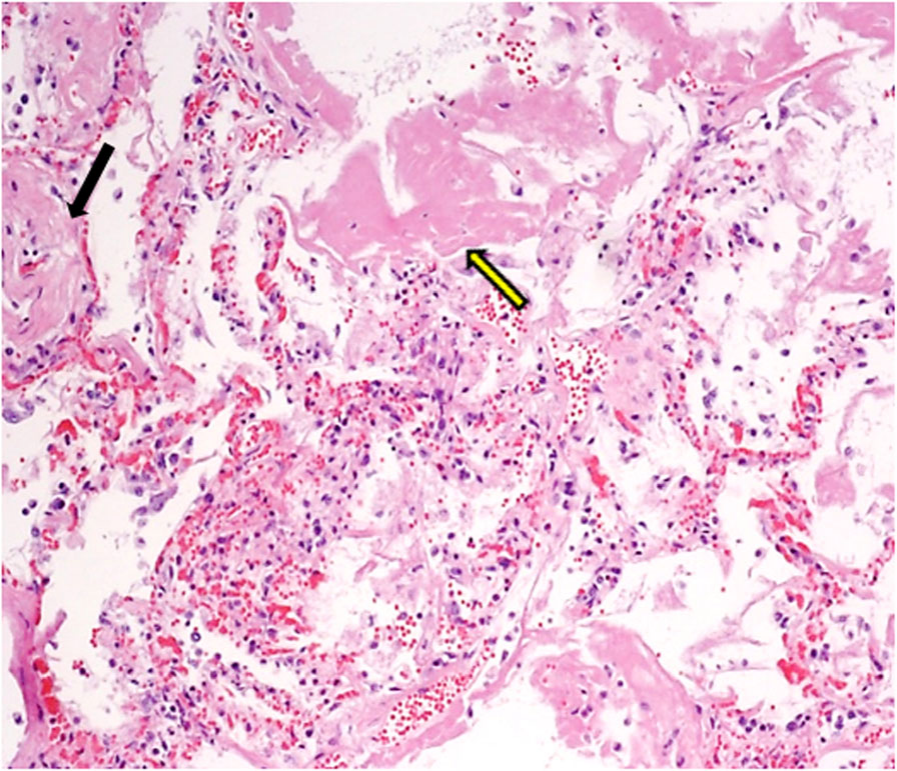
DAD pattern with hyaline membrane and moderate fibrotic organisation (black arrow); presence of scattered large protein globules (yellow arrow). Expanded alveolar septa with inflammatory cells (hematoxylin and eosin stain, × 200)

Myocardial vascular microthrombosis (Fig. 2). Diffuse alveolar damage (DAD) with organised hyaline membranes, sometimes with fibrotic aspects; presence of scattered large protein globules (Fig. 3). Expanded alveolar septa with inflammatory infiltrate (prevalently of lymphocytes and macrophages), fibroblasts and type II hyperplastic pneumocytes.

## Discussion

The two cases presented different stages of pulmonary morbidity linked to COVID-19. Respiratory viruses can cause a wide spectrum of pulmonary diseases [7]: in case 1, the exudative phase of acute interstitial pneumonia was observed indicating acute lung injury (ALI), while case 2 exhibited a later phase of DAD, with the development of ARDS. The histological findings correlated with the period of manifestation of symptoms: in case 1, the subject met with an early death because of the onset of acute coronary syndrome due to thrombosis of the anterior descending artery related to COVID-19 infection [8]; in case 2, the continuation of the infection caused a pulmonary thromboembolism and, in the lungs, a proliferative phase with prominent hyaline membranes and moderate fibrotic organisation.

Our subjects were non-oncological individuals, free of serious diseases and not hospitalised. Therefore, these are absolutely “pure” cases of COVID-19 infection, with anatomopathological patterns at different stages, not influenced by any other particular conditions.

It has been shown that defective lung architecture from mechanical tumour obstruction or previous lung surgery may also be predisposed to infection. Changes in the anatomy of the airways and lung tissue lead to the alteration of the intra-tumour microenvironment, which can secondarily influence the infiltration of immune cells. An increase in macrophages and inflammation carries an increased risk of cytokine release, which is a precursor to ARDS development [9]. Finally, it must be taken into account that not all patients react in the same way; some authors [10] indicate that the course of COVID-19 is not necessarily ominous in the presence of a compromised immune response and tend to reinforce the emerging therapeutic concepts of a controlled mitigation of the immune cascade following COVID-19 infection.

In the light of this evidence, the following conclusive considerations can be put forward with the aim of providing a better diagnostic-therapeutic framework for cancer patients infected with COVID-19:

a) Patients with severe clinical conditions are unlikely to present histopathological alterations of advanced phases of DAD: damage to such an extent occurs over a considerable period of time, but a patient with a poor clinical prognosis might not survive long enough for the DAD to become completely manifest.

b) An advanced anatomopathological pattern of DAD, however, could be related to the presence of lung cancer and the results of any chemotherapy treatments or surgery.

c) Although immunocompromised patients are more likely to contract the infection, the COVID-19 course is not always predictable in the presence of a compromised immune response.

d) Nosocomial infections other than COVID-19, responsible for the onset of ARDS, should not be ruled out for hospitalised patients.

## Data Availability

The datasets used during the current study are available from the corresponding author on reasonable request.

## Abbreviations

COVID-19: Corona Virus Disease 2019
ARDS: Acute respiratory distress syndrome
SARS-CoV-2: Severe acute respiratory syndrome coronavirus 2
RNA: Ribonucleic acid
PCR: Polymerase chain reaction
ALI: Acute lung injury
DAD: Diffuse alveolar damage.
